# Anti-Cancer Effects of Cyclic Peptide ALOS4 in a Human Melanoma Mouse Model

**DOI:** 10.3390/ijms22179579

**Published:** 2021-09-03

**Authors:** Bar Levi, Shiri Yacobovich, Michael Kirby, Maria Becker, Oryan Agranyoni, Boris Redko, Gary Gellerman, Albert Pinhasov, Igor Koman, Elimelech Nesher

**Affiliations:** 1 Department of Molecular Biology, Faculty of Natural Sciences, Ariel University, Ariel 4070000, Israel; barsh@ariel.ac.il (B.L.); shiriya@ariel.ac.il (S.Y.); michael.kirby566@gmail.com (M.K.); oryanag@ariel.ac.il (O.A.); albertpi@ariel.ac.il (A.P.); 2 Adelson School of Medicine, Ariel University, Ariel 4070000, Israel; mariabe@ariel.ac.il; 3 Department of Chemical Sciences, Faculty of Natural Sciences, Ariel University, Ariel 4070000, Israel; borisr@ariel.ac.il (B.R.); garyg@ariel.ac.il (G.G.); 4 Institute for Personalized and Translational Medicine, Ariel University, Ariel 4070000, Israel; igorko@ariel.ac.il

**Keywords:** cancer, cyclic peptide, integrin, α_v_β_3_, ALOS4, melanoma

## Abstract

We examined the effects of ALOS4, a cyclic peptide discovered previously by phage library selection against integrin α_v_β_3_, on a human melanoma (A375) xenograft model to determine its abilities as a potential anti-cancer agent. We found that ALOS4 promoted healthy weight gain in A375-engrafted nude mice and reduced melanoma tumor mass and volume. Despite these positive changes, examination of the tumor tissue did not indicate any significant effects on proliferation, mitotic index, tissue vascularization, or reduction of αSMA or Ki-67 tumor markers. Modulation in overall expression of critical downstream α_v_β_3_ integrin factors, such as FAK and Src, as well as reductions in gene expression of *c-Fos* and *c-Jun* transcription factors, indirectly confirmed our suspicions that ALOS4 is likely acting through an integrin-mediated pathway. Further, we found no overt formulation issues with ALOS4 regarding interaction with standard inert laboratory materials (polypropylene, borosilicate glass) or with pH and temperature stability under prolonged storage. Collectively, ALOS4 appears to be safe, chemically stable, and produces anti-cancer effects in a human xenograft model of melanoma. We believe these results suggest a role for ALOS4 in an integrin-mediated pathway in exerting its anti-cancer effects possibly through immune response modulation.

## 1. Introduction

Integrin α_v_β_3_ has been shown to play an essential role in different stages of cancer progression [1], metastasis [2], invasion [3,4], and angiogenesis [5]. Structurally, integrin α_v_β_3_ possesses a common integrin-binding motif and an Arg-Gly-Asp (RGD) recognition sequence [6] shared with several extra-cellular matrix (ECM) proteins including vitronectin, fibronectin, and fibrinogen [7]. Due to high expression in activated proliferating and angiogenetic [5] endothelial cells, integrin α_v_β_3_ has become a cancer theraputic target [8] and is considered a cancer prognostic biomarker [9] that correlates well with tumor progression [10,11] and invasion in such cancers as glioma [12], prostate carcinoma [13,14], osteosarcoma [2], breast cancer [9,15], and melanoma [16]. Melanoma is known to be one of the most fatal types of skin cancer, with a five-year relative survival rate of less than 20% for patients diagnosed with active metastasis [17,18]. Current therapeutic approaches to treatment of malignant melanoma include surgical resection of the tumor, immunotherapy, biological therapy, chemotherapy, radiation therapy, and combination targeted therapy [19]. The search for new therapeutic targets for a melanoma cure has revealed that overexpressed integrin α_v_β_3_ in transformed melanocytes [16] mediates tumor angiogenesis and is associated with organ-specific metastasis of human malignant melanoma [16], which has suggested a number of therapeutic approach possibilities for targeting α_v_β_3_. Among the approaches [20] used to inhibit integrin signal transduction, tumor growth, angiogenesis, and metastasis are blocking α_v_β_3_ with monoclonal antibodies [21], cyclic RGD antagonist peptides [22], or other antagonists [8]. Unfortunately, despite demonstrated anti-cancer activity in nude mice, previous attempts for developing α_v_β_3_ inhibitors such as the cyclic peptide Cilengitide [23] and functional anti-α_v_β_3_ antibodies such as Abegrin [24] have failed in clinical trials.

ALOS4, a synthetic 9-amino acid cyclic non-RGD peptide (NH_2_-CSSAGSLFC-COOH (MW = 871.98)) was previously discovered using a phage–display technique targeted to integrin α_v_β_3_ binding [25,26]. Using a murine melanoma model, we previously demonstrated anti-cancer properties of ALOS4 [25]. In this study, we investigated the effects of ALOS4 on a subcutaneous xenograft model of A375 human melanoma for effects on tumor growth, tumor tissue development, and expression of downstream targets of α_v_β_3_. In addition, we also characterized the physiochemical aspects of ALOS4 formulated stability and toxicity issues such as alterations in mouse behavior, blood cell profile, and blood chemistry in healthy (nominally cancer-free) mice. Our findings suggest that ALOS4 is stable in chemical formulation and poses no overt toxicity risks, yet is effective in melanoma tumor reduction by an α_v_β_3_-related mechanism and perhaps other mechanisms.

## 2. Results

### 2.1. ALOS4 Selectively Affects Tumor Development in the A375 Xenograft Model

**Figure 1:**
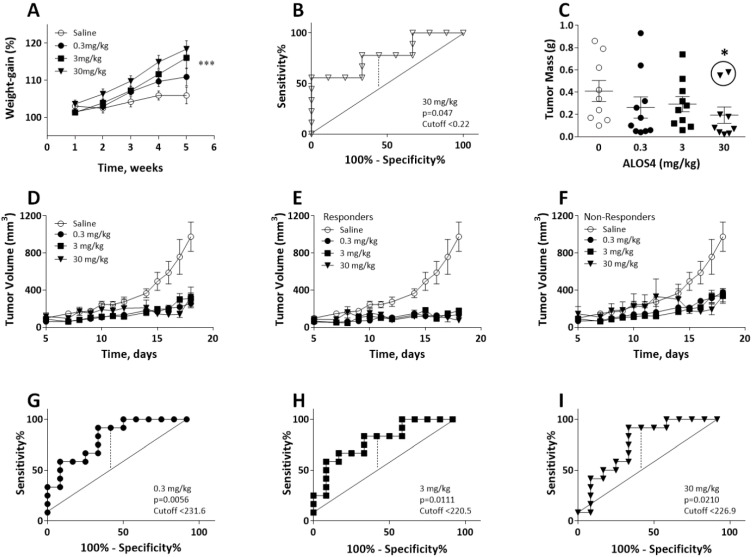
Effect of ALOS4 on body weight and tumor volume in SC A375 human melanoma mouse model. (**A**) Differences in the body weight gain of the nude mice inoculated with xenograft A375 SC tumor after 5 weeks administration with 0.3, 3, or 30 mg/kg of ALOS4. (**B**) ROC analyses of 30 mg/kg ALOS4-treated mice to determine threshold for positive drug response (Youden’s Index) at day 18 (*n* = 10). (**C**) Tumor mass (g) with two excluded (circled) non-responder mice based on ROC cutoff value. *, Dunn’s test *p* < 0.05. (**D**–**I**) ROC Analysis of responders and non-responders to ALOS4 treatment in SC A375 model. (**D**) Tumor volume growth in all treated nude mice. (**E**) Saline and ALOS4-treated responders only. (**F**) Saline and ALOS4-treated non-responders only. (**G**–**I**) ROC analyses of ALOS4-treated mice to determine threshold for positive drug response (Youden’s Index). * *p* < 0.05; *** *p* < 0.001. (*n* = 8).

In our previous research, we have shown that ALOS4 treatment leads to tumor growth inhibition and increased survival of C57BL/6J mice inoculated with murine B16F10 melanoma cells. In this study, we used a xenograft model to further confirm ALOS4 anti-cancer properties using immunodeficient nude mice, which were SC inoculated with human A375 melanoma cells followed by administration with 0.3, 3, or 30 mg/kg ALOS4. We found that 3 and 30 mg/kg of ALOS4 preserved normal weight gain of nude mice compared with untreated control animals, whose weight was significantly decreased during tumor development (two-way ANOVA followed by a Bonferroni means separation test: Interaction between weight and time F[39,490] = 0.5072, *p* = 0.9947; time F[30,490] = 37.52, *p* < 0.0001; treatment F[3,490] = 17.47, *p* < 0.0001; Figure 1A). A ROC analysis of tumor mass in mice treated with 30 mg/kg ALOS4 (Figure 1B) yielded a Youden’s index cut-off value of 0.22 (*p* = 0.047), which differentiated between responder and non-responder individuals, excluding two animals from analysis (Figure 1C, circled). Tumor mass data from lower doses of ALOS4 when analyzed by ROC did not yield significant results. Comparison of tumor mass collected at termination point at day 18 (not including two non-responders) demonstrated a dose-dependent inhibition of tumor growth by ALOS4 treatment (Figure 1C; Kruskal-Wallis ANOVA followed by a Dunn’s test, *p* = 0.0239).

We also observed that ALOS4 in a dose-dependent manner inhibited tumor growth (by estimated volume) in all examined concentrations showing maximal two-fold changes in growth inhibition with 30 mg/kg on day 17 (Figure 1D; two-way ANOVA followed by a Bonferroni means separation test: Interaction: F[33,310] = 3.590, *p* < 0.0001; Day: F[11,310] = 13.71, *p* < 0.0001; Treatment: F[3,310] = 42.13, *p* < 0.0001). We similarly conducted an ROC analysis of the results to distinguish responders from non-responders (Figure 1G–I). Responders (Figure 1E) and non-responders (Figure 1F) for each ALOS4 dose both yielded significant reductions in tumor volume compared with saline-injected control mice (two-way ANOVA followed by Bonferroni means separation test: Figure 1E: Interaction: F[33,156] = 2.860, *p* < 0.0001; Day: F[11,156] = 1.898, *p* = 0.0433; Treatment: F[3,156] = 36.04, *p* < 0.0001; Figure 1F: Interaction: F[33,202] = 1.809, *p* = 0.0072; Day: F[11,202] = 9.847, *p* < 0.0001; Treatment: F[3,202] = 20.43, *p* < 0.0001). Youden’s index values for ROC analysis of each ALOS4 dose were as follows: 231.6, *p* = 0.0056 (Figure 1G), 220.5, *p* = 0.0111 (Figure 1H), 226.9, *p* = 0.0210 (Figure 1I). TGI% values for each ALOS4 treatment group were similar and were as follows (ALOS4 mg/kg): 0.3, 61.1; 3, 66.3; 30, 61.5.

We further performed immunohistochemistry staining in ex vivo tumors obtained from SC xenografts to identify the effects of ALOS4 treatment on common hallmarks of cancer development and progression. Pleomorphism grades did not differ among control and ALOS4 treatments (all were rated at 2) and all examined tissue sections, regardless of treatment, had evidence of vascular invasion of the tumor mass (except tumor samples from one individual treated with 30 mg/kg ALOS4). Mitotic indices were also similar between controls and ALOS4 treatments (ALOS4 mg/kg, mean ± SD: 0, 6.2 ± 0.51; 0.3, 5.3 ± 1.13; 3, 6.68 ± 0.67; 30, 5.72 ± 0.98).

**Figure 2:**
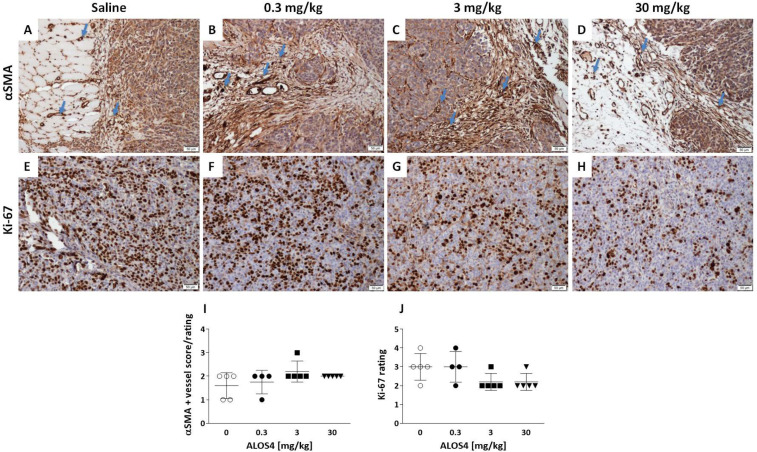
Effect of ALOS4 on carcinogenesis markers presentation in human melanoma A375 SC tumors from nude mice. (**A**–**D**) Representative photographs of slides stained for αSMA marker showing the number of blood vessels in the tissue sections (10×). Arrows demonstrate representative vessels in the tumor tissues. Scale: 50 µm. (**A**) Tumor of a saline-treated mouse shows low to moderate αSMA expression. (**B**) Tumor of an ALOS4 0.3 mg/kg-treated mouse demonstrates relative moderate vascular density around and within the tumor tissue. (**C**) Tumor of an ALOS4 3 mg/kg-treated mouse demonstrates relative moderate to high vascular density around and within the tumor tissue. (**D**) Tumor of an ALOS4 30 mg/kg-treated mouse demonstrates relative moderate vascular density around and within the tumor tissue. (**E**–**H**) Representative photographs of slides stained for Ki-67 marker. (**E**) Tumor of a saline-treated mouse shows a high number of positive cells within the neoplastic cell population. (**F**) Tumor of an ALOS4 0.3 mg/kg-treated mouse demonstrates a high number of positive cells within the neoplastic cell population. (**G**) Tumor of an ALOS4 3 mg/kg-treated mouse demonstrates a moderate to low number of positive cells within the neoplastic cell population. (**H**) Tumor of ALOS4 30 mg/kg-treated mouse demonstrates a moderate to low number of positive cells within the neoplastic cell population. Scale: 50 µm. (**I**,**J**) Quantification of histopathological evaluation scoring grades for αSMA (**I**) and Ki-67 (**J**) markers.

Analysis of the effect of ALOS4 on the expression of alpha smooth muscle actin (αSMA), a marker of vascular smooth muscle cells, was used to assess the number of blood vessels in the tissue sections to indicate the vascular invasion (Figure 2A–D). Controls treated with saline showed relatively low to moderate vascular density around and within the tumor tissue (Figure 2A). ALOS4 treatments of 0.3 and 30 mg/kg similarly showed moderate vascular density around and within the tumor tissue (Figure 2B,D), whereas ALOS4 treatment of 3 mg/kg showed relative moderate to high vascular density around and within the tumor tissue (Figure 2C). Overall, ALOS4 did not appear to produce any significant effects on tumor vascularization in the xenograft model of human melanoma (Figure 2I).

Non-parametric Kruskal–Wallis ANOVA analysis of Ki-67 proliferation marker showed a tendency toward dose-dependent reduction of expression in tumors treated with ALOS4 (*p* = 0.089). Thus, ALOS4 0.3 mg/kg dose and saline-treated controls both appeared to have a higher score in Ki-67-positive cells within the neoplastic cell population (Figure 2E,F). ALOS4 treatment with 3 mg/kg demonstrated a moderate to low number of Ki-67-positive cells (Figure 2G), whereas ALOS4 treatment with 30 mg/kg demonstrated a relatively low number of Ki-67-positive cells (Figure 2H). Comparisons of the Ki-67 results were performed using a tumor pathology scoring index for clinical relevance; however, and despite the appearance of dose-dependent reductions in Ki-67 expression, these reductions are not considered clinically meaningful.

### 2.2. The Effect of ALOS4 on Integrin-Related Signal Transduction

Since ALOS4 was discovered based on α_v_β_3_ integrin binding, we analyzed the effect of ALOS4 on integrin-related signal transduction. Integrin mediated “outside-in” signals, activate growth factor receptors and cytoplasmic kinases, which regulate gene expression of immediate early genes [27]. Activation of α_v_β_3_ integrin is known to induce the Fyn/Ras/Raf/MEK/ERK cascade, also called the MAPK pathway [28]. This pathway is highly or constantly activated in most cancer types and contributes to cancer proliferation, survival and migration [29]. Since we showed previously that ALOS4 treatment in B16F10 cells reduced migration [25], we hypothesized that ALOS4 may affect the MAPK pathway.

**Figure 3:**
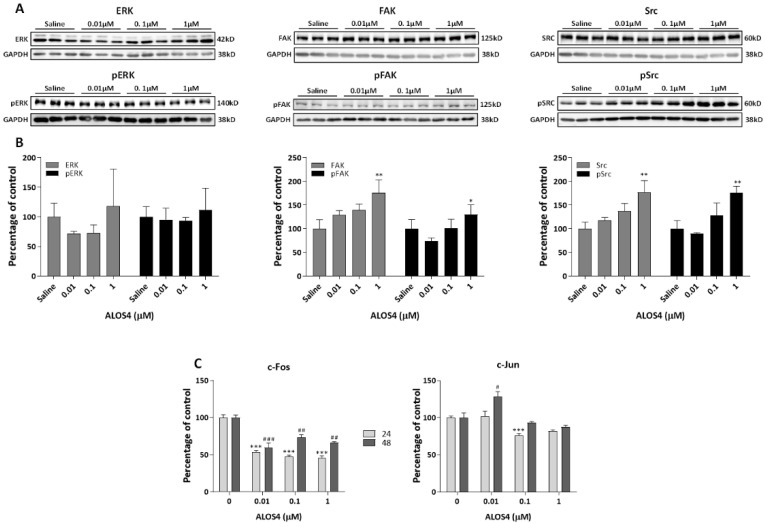
Effect of ALOS4 on α_v_β_3_ integrin signaling. Representative gel bands (**A**) and Western blot densitometry results (**B**) performed for A375 cells treated for 48 h with ALOS4 at 0.01, 0.1, or 1.0 µM and analyzed for ERK/pERK, FAK/pFAK, and Src/pSrc protein expression. Data are presented as percentage of control normalized to GAPDH, *n* = 3 for each treated group. (**C**) A375 cells treated for 24 (*n* = 6) and 48 (*n* = 3) h with ALOS4 at 0.01, 0.1, or 1.0 µM were analyzed for *c-Fos* and *c-Jun* mRNA expression using qRT-PCR. Data presented as percentage of control. */# at *p* < 0.05, **/## at *p* < 0.01, and ***/### at *p* < 0.0001.

A375 cells were treated with concentrations of 0.01, 0.1, or 1.0 µM for 48 h and protein extracts were prepared for Western blots. We observed that 1.0 µM of ALOS4 significantly upregulated focal adhesion kinase (FAK), as well as proto-oncogene tyrosine protein kinase (Src) and pSrc levels (Figure 3A,B; One-way ANOVA: FAK, F[3,8] = 12.86, *p* = 0.0020; Src, F[3,8] = 12.1, *p* = 0.0024; pFAK, F[3,8] = 5.28, *p* = 0.0267; pSrc, F[3,8] = 14.44, *p* = 0.0019), while not affecting levels of extracellular signal-regulated kinase (ERK) and pERK (ERK, F[3,8] = 1.288, *p* = 0.3429; pERK, F[3,8] = 0.3928, *p* = 0.7617).

We also examined the expression of the immediate early genes *c-Fos* and *c-Jun* in ALOS4-treated A375 human melanoma cells. A375 cells were treated with ALOS4 at concentrations of 0.01, 0.1, or 1.0 µM for 24 h or 48 h and RNA was extracted for qRNA analysis. We found that ALOS4 treatment significantly decreased *c-Fos* gene expression after 24 and 48 h (Figure 3C, left panel; One-way ANOVA: 24 h, F[3,20] = 76.99, *p* < 0.0001; 48 h, F[3,8] = 19.19, *p* = 0.0005). A similar phenomenon was observed in *c-Jun* transcription levels, which showed significant decrease after 24 h at higher doses (Figure 3C, right panel; One-way ANOVA: F[3,15] = 10.69, *p* = 0.0005), whereas *c-Jun* was increased by 0.01 µM ALOS4 at 48 h (Figure 3C, right panel; One-way ANOVA: F[3,5] = 9.331, *p* = 0.0172).

### 2.3. ALOS4 Does Not Adhere to Inert Materials and Is Stable over a Range of Acid/Base and Temperature Conditions

**Figure 4:**
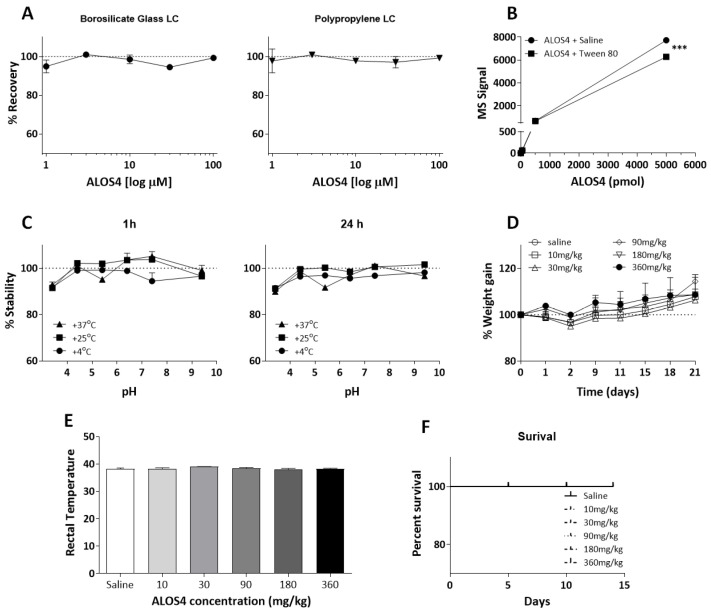
ALOS4 inert materials adherence, pH stability, temperature stability, safety, and toxicity. (**A**–**C**) LC-MS analysis of ALOS4 recoverability following inert materials exposure and storage under different pH, temperature, and formulation time conditions. (**A**) ALOS4 does not adhere to borosilicate LC glass vials or polypropylene LC vials at concentrations up to 100 µM. Data represent mean ± SEM (*n* = 3). (**B**) ALOS4 showed optimal stability in standard saline solution (0.9% NaCl) in comparison with saline containing 0.1% Tween-80 at concentrations of 0.5, 5, 50, 500, or 5000 pmol/µL ALOS4 incubated for 60 min at 25 °C in standard polypropylene tubes. Data represent mean ± SEM (*n* = 3), *** at *p* < 0.001. (**C**) ALOS4 demonstrates stability in a variety of pH and temperature conditions. ALOS4 (10 µM) was incubated in standard polypropylene tubes under different pH conditions at and stored at 4, 25, or 37 °C for 1 or 24 h. Data represent mean ± SEM (*n* = 3). (**D**,**E**) ALOS4 shows no effect on ICR mouse (**D**) body weight or (**E**) body-temperature following repeated IV administration at 10, 30, 90, 180, or 360 mg/kg ALOS4 (*n* = 5). Weight-gain of mice was measured three times per week, one hour prior to ALOS4 injections. Percent of weight change was calculated according to baseline weight prior to treatment. Body temperature was measured 30 min after ALOS4 administration. (**F**) ALOS4 repeated doses (10, 30, 90, 180, or 360 mg/kg) administrated intravenously for 14 days did not affect survival of ICR mice (*n* = 5).

We chose to perform a series of chemical stability and recoverability tests on ALOS4 to determine its practical applicability as a drug in formulation. To ensure that ALOS4 was stable and did not adhere to standard laboratory materials, we incubated ALOS4 formulated in 0.9% NaCl solution at a range of concentrations from 1–100 µM in polypropylene microtubes for 60 min at room temperature, then transferred solutions to either polypropylene or borosilicate glass liquid chromatography (LC) vials. LC-MS analysis showed that ALOS4 recoverability was near 100% in both borosilicate LC glass vials and standard polypropylene LC vials (Figure 4A).

Solubilizing agents are commonly used to stabilize peptides in solution and to reduce inert substrate interaction. Therefore, we compared two solvent options for ALOS4, standard saline solution (0.9% NaCl) and saline solution containing 0.1% Tween-80 (polysorbate). Solutions of ALOS4 ranging from 0.5–5000 pmol/µL formulated in both solvents were incubated for 60 min at room temperature in standard polypropylene tubes. LC-MS analysis showed that ALOS4 formulated in the saline solution containing Tween-80 had significantly reduced peptide stability by 1.2-fold in comparison with ALOS4 formulated in 0.9% NaCl only (Figure 4B; Two-way ANOVA, F(1,10) = 3217.54, *p* < 0.0001).

Evaluation of ALOS4 (10 µM) stability at different ranges of acid/base and temperature conditions was conducted in saline after 1 or 24 h in different pH solutions above and below the physiological pH (7.4): pH 3.4, 4.4, 5.4, 6.4, 7.4, and 9.4. Stability analysis was also performed under three regimes: at 4 °C (storage temperature), 25 °C (room temperature), and 37 °C (body temperature). LC-MS analysis indicated that ALOS4 was highly stable (90–100% recoverability) at all measured temperatures and all analyzed acid/basic conditions at both time points (Figure 4C).

### 2.4. ALOS4 Shows High Safety and No Toxicity In Vivo

To evaluate ALOS4 safety and toxicity, uninoculated (nominally cancer-free) ICR mice were IV administrated ALOS4 and monitored for clinical signs of toxicity including body weight changes, body temperature, alopecia, nasal bleeding, and mortality. ICR mice were injected with doses of ALOS4 every other day over 21 days with 10, 30, 90, 180, or 360 mg/kg (for a total of ten doses). Mouse weights were taken daily and rectal temperatures were recorded 30 min following ALOS4 injection. We observed no alterations in weight-gain (Figure 4D) or body temperature changes (Figure 4E) of treated mice in comparison with control mice during the course of the trial. Further, to determine the maximal tolerant dose (MTD) of ALOS4, ICR mice were ALOS4 IV-injected at a range of doses from 10–360 mg/kg and monitored for survival and clinical symptoms. We found that even at the maximum tested repeated dose of 360 mg/kg ALOS4, 21-day survival was 100% (Figure 4F). No adverse overt clinical signs were observed during the trial. Necropsy assessment for organ damage (histology of liver, spleen, kidney, lungs, and brain) did not reveal any overt signs of tissue damage or inflammation.

To evaluate potential effect of ALOS4 on mouse locomotory activity and anxiety-like behaviors, we used two standard behavioral paradigms: the open-field ambulation and elevated plus maze (EPM) tests.

**Figure 5:**
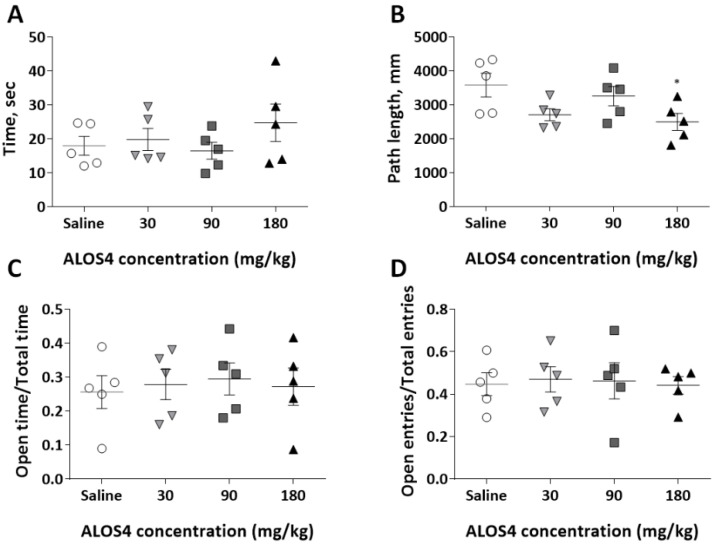
ALOS4 does not affect mouse locomotion or produce anxiety-like behaviors. (**A**,**B**) Behavior of ICR mice (*n* = 5) was not affected by intravenous administration with three acute single doses of ALOS4 (30, 90, or 180 mg/kg). Mice tested in the open-field arena for locomotory activity showed no changes in either cumulative central arena area dwell time (**A**) or total traveled distance, * at *p* < 0.05 (**B**). Mice tested in the EPM test for anxiety-like behavior showed no changes in two analyzed parameters (with exception to 180 mg/kg ALOS4 for OE/TE ratio): Open arm time (OT):/Total time (TT) ratio (**C**); Open entry (OE)/Total entry (TE) ratio (**D**).

We found that an IV acute single dose of ALOS4 of 30, 90, or 180 mg/kg administered to ICR mice did not affect locomotor activity in general (Figure 5A: One-way ANOVA analysis, F[3,16] = 0.9406, *p* = 0.4441) with only an exception for total traveled distance at the dose of 180 mg/kg (Figure 5B: One-way ANOVA, F[3,16] = 3.308, *p* = 0.0471, followed by Bonferroni’s means separation test [180 mg/kg, *p* = 0.0329]), and did not produce any anxiety-like behaviors (Figure 5C: One-way ANOVA, F[3,16] = 0.1064, *p* = 0.9551; Figure 5D: One-way ANOVA, F[3,16] = 0.04374, *p* = 0.9874).

### 2.5. ALOS4 Does Not Affect Blood Cell Counts or Blood Chemistry

Blood of ICR mice collected 24 h after ALOS4 IV treatment with acute single doses of 30, 90, or 180 mg/kg and was evaluated for complete blood count (CBC) and basic blood chemistry profile. Analysis of blood compared with the normal rage of ICR mice blood scores [30] showed that ALOS4 generally does not affect blood counts of ICR mice when compared with the control group injected with saline. There were several cell count values that differed from the established laboratory normal range with some doses of ALOS4 treatment; however, control mice treated with saline also deviated from the laboratory normal values as well. Specifically, in the CBC (Table 1), lower values were observed in white blood cells (WBC), mean corpuscular hemoglobin (MCHC), and platelet counts (except 90 mg/kg dose) of ALOS4-treated animals. However, to attribute this decrease to an ALOS4-specific effect may not be correct, since in most of these cases the saline-treated mice also had scores laying out of normal range and may simply be an injection response (Table 1). We observed a 40% decrease in WBC count with 180 mg/kg ALOS4, which was far outside the normal range. Blood biochemical results of ALOS4 and control-treated mice showed higher than normal or normal values in cholesterol, TP, and alkaline phosphate concentrations and lower than normal range in both total bilirubin and chlorides in all injected groups (Table 2). Thus, ALOS4-dependent alterations of blood parameters were minor and clinically non-significant in comparison with controls.

## 3. Discussion

α_v_β_3_ integrin is an important cell adhesion receptor involved in various biological activities [18,31] acting through cell signal transduction from the cell membrane to several cytosolic pathways [27]. Due to overexpression of α_v_β_3_ in many cancers [21], this integrin is a desirable therapeutic target for cancer treatment. Several anti-cancer peptides [23,32] were developed for α_v_β_3_ inhibition targeting the Arginine-Glycine-Aspartate (RGD) motif. Despite their promising potential, these peptides failed in late clinical trials [33], possibly due to their competitive binding to the ECM proteins, which also have RGD sites [34]. In this work, using A375 human melanoma cells we demonstrated anti-cancer properties of ALOS4, a non-RGD peptide thought to target the α_v_β_3_ integrin signaling pathway. This peptide was discovered in our laboratory using a phage display technique and previously demonstrated an anti-cancer efficacy in a murine melanoma model [25]. The potential of ALOS4 as a formulated drug is further demonstrated in its physical and chemical stability, as well as appearing to have a good safety profile.

Using a subcutaneous model of A375 human melanoma, we demonstrated high efficacy of ALOS4 in tumor growth inhibition during an 18-day trial. Since there were limitations regarding the number of mice permitted for study, thus also restricting the number of dosing groups, we applied receiver operating characteristic (ROC) analyses [35] and calculated Youden’s indices to account for result variability. This enabled us to distinguish between treatment responder and non-responder mice at examined doses of ALOS4 and helped to explain the observed group variability (i.e., segregated responder and non-responder mice were highly internally consistent in their responses to treatment). The existence of “non-responders” is a phenomena extensively discussed in the medical literature describing cancer patients who do not respond to conventional therapies [36,37] and can be explained by variability in the patient microbiome [38] and differential expression of cancer cell surface proteins [39]. These explanations may also be applicable to ALOS4 treatment non-responders observed in our experiments. However, such extrapolation requires further characterization to account for the mechanistic basis of differential treatment responses to ALOS4. Determining the underlying mechanism of ALOS4 has allowed us to eliminate a few possibilities. For example, ex vivo analysis of xenograft tumors did not indicate any significant effect of ALOS4 on angiogenesis or proliferation rates, despite the ALOS4 dose-dependent decrease in tumor size observed. Finally, it is interesting to mention that mice treated with ALOS4 did not lose body weight in contrast with untreated animals. In fact, mice treated with ALOS4 even gained weight leading us to believe that ALOS4 may be used for treatment of cancer patients at different stages of disease suffering from cachexia [40,41], which is considered in 20–40% of cases as an immediate cause of death [42,43].

Since ALOS4 was developed as an α_v_β_3_ integrin-targeted molecule, it is likely that observed anti-cancer effects were achieved through modulation of α_v_β_3_ integrin signaling. To confirm this suggestion, we analyzed changes in expression of selected candidates from the α_v_β_3_ integrin signaling pathway, including extracellular signal-regulated kinases (ERK; also known as mitogen-activated protein kinases or MAPK) initiated by activation of focal adhesion kinases (FAK) and Src kinases, which in complex or individually further activate downstream ERK signaling [44,45]. We found that ALOS4 does not alter total ERK, despite significant upregulation of FAK, or alter Src protein expression at high doses in human melanoma cells in vitro. Since ALOS4 alters FAK and Src, but not ERK which acts as the last messenger of MAPK/ERK pathway prior to entering the nucleus and activating transcription factors of genes involved in proliferation and metastasis [46], we suggest that additional modulation occurs interrupting downstream signals. Furthermore, the final products of ERK signaling, the oncogenes *c-Fos* and *c-Jun*, were both affected by ALOS4 treatment in vitro. ALOS4 significantly reduced *c-Fos* mRNA levels at all doses, while downregulation of the *c-Jun* gene was significant at 0.1 µM dosage after 24 h and showed a non-significant tendency to decrease after 48 h. We speculate that the differences in levels of significance in *c-Fos* expression at 24 and 48 h of treatment may indicate the attempt of the cancer cells to stabilize expression of this oncogene, whereas its downregulation by ALOS4 remains to be explained. These results indirectly confirm that ALOS4 is able to modulate α_v_β_3_ integrin signaling and differences in the effect of ALOS4 on *c-Fos* and *c-Jun* expression may be explained by additional ALOS4-independent processes involved in *c-Fos* and *c-Jun* transcription. The *c-Fos* results are not without precedent considering the actions of other peptide-based integrin antagonists (flavoridin) in melanoma cell lines, which increase activation of downstream integrin pathway elements (such as increased FAK phosphorylation) while also effecting downregulation *c-Fos* expression [47]. Thus, since ALOS4 was developed targeting α_ν_β_3_ integrin and its ability to bind α_ν_β_3_ leading to metastatic arrest was previously demonstrated [25], we believe that our new results indirectly confirm the involvement of ALOS4 in the modulation of selected components of integrin signaling. However, unaffected ERK in the presence of upregulated FAK and Src suggests that an additional intervening pathway modulating ERK-related signaling, possibly through integrin-initiated RAS-RAF activating cascade [48,49] or integrin independent signaling pathway [50], is present and a further study of molecular mechanisms of action at a higher-resolution with ALOS4 is required.

We also examined ALOS4 safety and stability as a potential drug candidate. Due to a known tendency of peptides to adhere to standard inert laboratory materials [51], we demonstrated that regardless of ALOS4 concentration, we achieved nearly 100% peptide recovery in both analyzed materials (borosilicate LC glass vials and standard polypropylene LC vials). We posit that the allosterically-constrained, cyclical structure of the peptide may be the reason ALOS4 does not significantly interact with typically problematic laboratory materials as do other peptides. Furthermore, despite the fact that most peptides in solution undergo degradation by hydrolysis or oxidation [52], ALOS4 was highly stable in saline solution over a wide range of acid/base conditions at different temperatures, features which are beneficial for long-term storage and ease-of-use for therapeutic applications [53]. We also examined the toxicity of ALOS4 in nominally cancer-free mice, which is considered an essential factor for pharmaceutical safety [54,55]. ALOS4 demonstrated no toxicity in vivo with repeated treatments over a range of doses from 10 to 360 mg/kg and no mortality or serious adverse events were observed. We also elected to examine whether repeated ALOS4 dosing would produce any unfavorable behavioral features, such as sedation, hyperactivity, or anxiety-like behaviors. No adverse behavioral effects were observed. Hence, when comparing with the therapeutic doses of other anticancer peptides, which range from 2.5 mg/kg (Cilengitide [56]) to 60 mg/kg (HM-3 [57]), ALOS4 stands out as a remarkably non-toxic compound. Moreover, whereas most conventional chemotherapies are accompanied by severe side effects that require medical intervention [58], the safety profile of ALOS4 shows potential as an anti-cancer drug that may be tolerable for patients.

Blood chemistry was also examined during toxicity studies and revealed reduction of white blood cell counts (40%) with ALOS4 treatment at 180 mg/kg. This reduction could indicate higher levels of tumor-infiltrating lymphocytes (TILs), suggesting that the immune system may be involved in the ALOS4 activity. Unfortunately, potential effects and mechanistic outcomes of immune interactions of ALOS4 could not be determined in this study due to the immunodeficient nature of the mice required for the xenograft model. Nevertheless, cumulative results from this work and prior studies suggest an interaction of ALOS4 with immune system elements, which needs to be further evaluated.

In summary, ALOS4 appears to be completely non-toxic, remarkably prolongs lifespan, and increases weight of treated mice. The latter feature makes ALOS4 beneficial to counteract cachexia experienced by cancer patients during the process of disease progression. We believe that demonstrating the anti-cancer activity through modulation of components of integrin signaling together with its safety profile suggests that ALOS4 peptide is a promising patient-tolerable prospective anti-cancer drug candidate.

## 4. Materials and Methods

### 4.1. ALOS4

ALOS4 was developed based on α_v_β_3_ binding using phage display technology. This synthetic cyclic peptide is composed of the following nine-amino-acid sequence: H-cycl(Cys-Ser-Ser-Ala-Gly-Ser-Leu-Phe-Cys)-OH. ALOS4 was custom-synthesized by Shanghai Hanhong Scientific Co. (Cat#P120301-LG221431, Shanghai, China). Stock solutions of ALOS4 at 10 mM, were prepared in sterile physiological saline solution (0.9%; Sigma-Aldrich, Cat#7647-14-5, Darmstadt, Germany) with the addition of 0.02% BSA (Biological Industries, Cat#1522089, Kibbutz Beit-Haemek, Israel) and maintained at either −20 °C for short term use, or −80 °C for long-term storage. For each experiment, ALOS4 was thawed and freshly diluted to working concentrations in physiological saline.

### 4.2. Cell Cultures

A375 human melanoma cells (ATCC; Cat#CRL-1619, Manassas, VA, USA) were grown in Dulbecco’s Modified Eagle Medium (DMEM; Fisher Scientific [Gibco], Cat#41965-039, Hampton, NH, USA) with 4.5 g/L glucose and L-glutamine, supplemented with 10% fetal bovine serum (FBS; Fisher Scientific [Gibco], Cat#16000-036, Hampton, NH, USA) and 1% penicillin-streptomycin (Fisher Scientific, Cat#10378-016, Hampton, NH, USA). Cells were maintained on uncoated dishes in atmosphere of 5% CO_2_ at 370 °C.

### 4.3. Chemical Properties Assays

Adhesiveness to inert materials was measured for ALOS4 0.9% NaCl(aq) solution in concentrations of 1, 3 10, 30, or 100 µM and incubated in standard laboratory polypropylene microtubes for 60 min at room temperature. Solutions were transferred to either polypropylene LC vials or borosilicate glass LC vials. Optimal formulation stability of ALOS4 was analyzed in either saline solution (0.9% NaCl) or saline solution containing 0.1% tween 80 (polysorbate; Sigma-Aldrich, Cat#P1754, Darmstadt, Germany) at concentrations of 0.5, 5, 50, 500, or 5000 pmol/µL. Solutions were incubated in standard polypropylene tubes for 60 min at room temperature. Acid/base stability was measured for ALOS4 formulated in saline at a concentration of 10 µM and incubated for 1 or 24 h at 3.4, 4.4, 5.4, 6.4, 7.4, or 9.4 pH at temperatures of 4, 25, or 37 °C. Recovery of ALOS4 following these materials assays was assessed by LC-MS.

### 4.4. Animals

To investigate the effect of ALOS4 on human melanoma cancer cells, nude Fox nu/nu mice were used for SC- or IV-injected inoculations. Additionally, uninoculated (nominally cancer-free) ICR mice were used for safety and toxicity studies. Mice were obtained from Envigo, Israel, and arrived at the age of 4–5 weeks old. Upon arrival, mice were habituated to vivarium conditions for one week before initiation of experiments. All mice were maintained under a 12:12 light–dark cycle and provided Purina rodent chow (Envigo, Ness-Ziona, Israel) and water ad libitum. Animals were housed five to a cage in a room maintained at 22 ± 0.5 °C (nude mice cages were held in a laminar-flow cabinet).

### 4.5. Behavioral Models

#### 4.5.1. Open Field

To evaluate the effect of ALOS4 on mouse locomotor activity, we used the open field (OF) behavioral test [59,60]. This assay consists of an arena (30 × 40 cm) with no grid markings and uses an infrared imaging system. The number of entries into the arena center zone was recorded using EthoVision 7.1 software (Noldus Information Technology, Wageningen, The Netherlands). Each mouse was placed individually in the center of the arena and evaluated for 6 min. Arena center dwell time versus arena border dwell time, as well as total traveled distance, were recorded. To provide a less stressful environment, the test was performed in a semi-dark room. One hour prior to the test, all mice were placed in the behavioral experiment room for acclimation. Between subjects, the apparatus was thoroughly washed with 70% ethanol and dried.

#### 4.5.2. Elevated plus Maze

To evaluate the effect of ALOS4 on mouse anxiety-like behaviors, we used the elevated plus maze test (EPM) [60]. The EPM consists of a plus-shaped arena with two open (10 × 45 × 40 cm) and two enclosed (10 × 45 × 40 cm) open-roof arms, elevated 70 cm from the floor. Each mouse was placed in the center of the maze and was free to move in the arena for 5 min. The number of entries into open and closed arms, as well as time spent in the open and closed arms (dwell time), was recorded using EthoVision 7.1 software (Noldus Information Technology, Wageningen, The Netherlands). To provide a less stressful environment, the test was performed in a semi-dark room. One hour prior to the test, all mice were placed in the behavioral experiment room for acclimation. Between subjects, the apparatus was thoroughly washed with 70% ethanol and dried.

#### 4.5.3. Toxicity Assessment

Acute single or repeated doses of ALOS4 at 10, 30, 90, 180, or 360 mg/kg were administered intravenously to uninoculated (nominally cancer-free) ICR mice. Body weight, rectal temperature, and survival were evaluated for 14 days following injections. Mice were also evaluated for locomotory and anxiety-like behaviors (open-field and EPM) on treatment day 14.

#### 4.5.4. Subcutaneous Model of Melanoma

We used a subcutaneous (SC) melanoma model to study the effect of ALOS4 on localized solid tumor growth. Nude mice (Fox nu/nu) were SC injected with A375 cells at 2 × 10^6^ cells in 100 μL in serum-free DMEM medium/mouse. Following inoculation, all mice were randomly divided into experimental groups, then treated IP with either ALOS4 or saline (negative control) at day one post-inoculation. Mouse body weights were monitored during the course of the experiment. The base-weight of mice was determined by the weight on the second day to account for acclimation-related changes, then mice were weighed twice a week until tumor appearance and thereafter daily until the experiment was terminated. Termination resulted from mouse death, when tumor diameter reached or exceeded 1500 mm^3^, or when 30 days of treatment had elapsed, whereupon mice were CO_2_ euthanized. Mice were IP-injected with ALOS4 (0.1, 0.3, or 30 mg/kg; assumed therapeutic range) or saline (control) at identical fixed volumes. SC inoculation of cells usually formed a palpable tumor in 7–14 days. Tumor volumes were estimated by digital caliper and calculated with the following equation: V(tumor, mm^3^) = π/6 × width × length × height. Survival rate of mice was documented at the end point of experiments. Tumor growth rates were calculated by the following formula: TGI% = (relative tumor volume ALOS4-treated)/(relative tumor volume saline-treated).

#### 4.5.5. Immunohistochemistry of A375 Tumor

Nude mice (Fox nu/nu) were SC-inoculated with A375 human melanoma cells (2 × 10^6^ cells in 100 μL normal saline/mouse) and treated for 18 days with ALOS4 (0, 0.3, 3, 30 mg/kg) injected IP with daily monitoring for clinical signs. Mice were euthanized by CO_2_ asphyxiation when the first mouse reached the ethical protocol limit of 1500 mm^3^ tumor size. Tumors were harvested, weighed, and fixed in 4% formalin. After 24 h fixation, samples were rinsed with PBS and transferred to 70% ethanol for transport to the pathology laboratory. Embedding, 5 μm sectioning, and slide preparation were performed for the 5 tumors from each experimental group (*n* = 4 for the 0.3 mg/kg due to the technical issues within processing) according to routine procedure.

Tumor pathology was rated by a certified veterinary pathologist (Patho-Logica, Rehovot, Israel) using the following scales when examined at 40× magnification: Pleomorphism (0, none; 1, mild; 2, moderate; 3, severe), mitotic index (mitotic indicators were counted in 10 different 40× fields and averaged), degree of vascular invasion (−, none; +, invasion). Prepared tumor tissue slides were also evaluated for the presence of tumor-related-markers by monoclonal antibody staining for the α-smooth muscle actin (αSMA), the marker of vascular smooth muscle cells, as well as the nuclear protein cell proliferation marker Ki-67 using the following rating scales: αSMA (0, not present; 1, mild [10–20 positive vessels]; 2, moderate [20–50 positive vessels]; 3, severe [>50 positive vessels]), Ki-67 (0, not present; 1, <10%; 2, 10–50%; 3, 50–75%; 4, >75%).

#### 4.5.6. RNA Extraction and qRT-PCR

RNA from A375 cells 24 and 48 h after the treatment was purified from cells using a quick RNA miniprep kit (Zymo Research, Cat#R1018, Irvine, CA, USA). DNase treatment was performed using on-column DNase digestion. RNA concentration was measured at 260 nm using NanoDrop spectrophotometer (Thermo Scientific, Wilmington, DE, USA, Cat#DE19810) and 260/280 ratio method was used to verify that the samples met proper purification standards around 2. A total of 1 μg of total RNA was reverse-transcribed using a reverse transcription system (Promega, Cat#A3500, Madison, WI, USA). The master mix for cDNA synthesis insisted of 10× Reverse Transcription buffer, dNTP mix, oligo (dT) (18T) primers, and AMV enzyme. The reverse transcription reaction was performed in a thermocycler (Bio-Rad Laboratories, T100, Hercules, CA, USA) using a two-step program: 42 °C for 60 min followed by heating to 70 °C for 15 min to terminate the reaction, and maintained at 4 °C. The quantitative RT-PCR for *c-Fos* and *c-Jun* was performed using 2× PCR SYBR Green Master Mix (Applied Biosystems, Cat#4344463, Warrington, UK), with a 100 nM mixture of forward and reverse primers (*c-Fos* forward: ctggcgttgtgaagaccat and reverse: tcccttcggattctcctttt; *c-Jun* forward: atcaaggcggagaggaagc and reverse: tgagcatgttggccgtggac; as well as HPRT used as an endogenous normalization factor, forward: cctggcgtcgtgattagtgat and reverse: tcgagcaagacgttcagtcc), 4 µg of cDNA and RNase/DNase free water. Samples were placed in Real-Time PCR (AriaMx; Cat#G88230A, Santa Clara, CA, USA,) and reactions were performed in a thermocycler: 180 s at 95 °C, followed by 40 cycles of 3 s at 95 °C and 30 s at 60 °C.

#### 4.5.7. Protein Extraction and Western Blot Analysis

Proteins from A375 cells were extracted in RIPA lysis buffer solution (150 mM NaCl, 50 mM Tris-HCl pH 8.0, 1% Triton X-100, 0.5% sodium deoxycholate, 0.1% SDS) with freshly added 1 mM sodium orthovanadate (Na_3_VO_4_; Sigma-Aldrich, Cat#S6508, Darmstadt, Germany), 5 mM sodium fluoride (NaF; Sigma-Aldrich, Cat#S7920, Darmstadt, Germany), protease inhibitor cocktail (Millipore, Cat#539134, Burlington, MA, USA), and phosphatase inhibitor (Sigma-Aldrich, Cat#4906845001, Darmstadt, Germany). Protein concentrations were assessed using a Bradford assay. Proteins were separated by gel electrophoresis in an 8% polyacrylamide gel. Blots were incubated with blocking solution (5% BSA in TBST: Bio-Lab, Cat#208923, Jerusalem, Israel) for 1 h with gentle shaking at room temperature. After blocking, separate membranes were each probed with one of target-specific antibodies (ERK1/2, Millipore, Cat#MABS827, Burlington, MA, USA; pERK, Cell Signaling, Cat#C33E10, Danvers, MA, USA; FAK, Cell Signaling, Cat#3285; pFAK, Santa Cruz, Cat#sc-374668, Dallas TX, USA; c-Src, Novus Biologicals, Cat#5A18, Littleton CO, USA; p-c-Src, Santa Cruz, Cat#sc-166860), then hybridized with horseradish peroxidase-conjugated streptavidin secondary antibodies (Abcam, Cat#ab6802 and ab205719, Cambridge, UK) and developed using ECL solution (Immobilon Crescendo Western HRP substrate, Millipore, Cat#ELLUR0100, Burlington, MA, USA) according to the manufacturer protocol. After the antibody of a specific protein on each membrane was evaluated, we performed a GAPDH (Millipore, Cat#MABS819, Burlington, MA, USA) re-probe for all of membranes to quantify the target proteins. FAK and Src were performed after stripping on the same membrane and that is why they share their common GAPDH. Blots were visualized using ChemiDoc™ Imaging System (Bio-Rad Laboratories, Hercules, CA, USA) apparatus and densitometry analysis was performed using Image Lab Software (Bio-Rad Laboratories, Hercules, CA, USA). The grouped data sets representing phosphorylated and total proteins demonstrates percentage of each treated group normalized to untreated control.

#### 4.5.8. Complete Blood Cell Count and Blood Chemistry

ICR mice were treated with single IV injections of ALOS4 (30, 90, or 180 mg/kg) and blood samples were collected at 24 h post-injection to EDTA and serum tubes. Complete blood cell count (CBC) and blood chemistry analyses were performed for saline and ALOS4 treatment groups at a certified animal laboratory (Herzliya Medical Center, Herzliya, Israel).

#### 4.5.9. Statistical Analysis

All data are expressed as means ± SE (±SD in a few measures). Threshold for significance was set to α = 0.05. Multiple treatments were Bonferroni-corrected and compared by unmatched one-way ANOVA for single time point results or by two-way ANOVA for multiple treatment outcomes over time. ANOVA tests were followed with a Bonferroni means separation test to identify specific differences between treatments. For ordinal or nominal data of tumor immunohistochemistry scoring, groups were compared by Kruskal–Wallis ANOVA followed by a Dunn’s post-test for intergroup comparisons [61]. To differentiate between responders and non-responders for tumor growth effects, we performed responder operator curve analyses (ROC) to separate groups. ROC analyses and ANOVAs including post-tests were performed with GraphPad Prism 7.0.

## 5. Patents

Pinhasov A. has an ALOS4 patent (62/127,854).

## Figures and Tables

**Table 1:** Complete blood count of ALOS4-treated ICR mice.

	Normal Range	Saline	ALOS4 30 mg/kg	ALOS4 90 mg/kg	ALOS4 180 mg/kg
**WBC 10^3^/µL**	6.5–24.5	5.49 ± 1.8	4.51 ± 1.3	6.07 ± 1.2	3.2 ± 0.06
**RBC 10^6^/µL**	7.31–10.03	9.21 ± 0.8	9.16 ± 0.3	9.78 ± 0.3	8.02 ± 0.6
**HGB g/dL**	13.1–16.2	14.68 ± 1.02	14.3 ± 0.6	15.42 ± 0.5	13.2 ± 1.04
**Hematocrit %**	36.8–48.7	44.8 ± 3.2	43.96 ± 1.9	47.88 ± 1.1	40.12 ± 3.15
**MCV fL**	46.0–50.9	48.94 ± 1.2	47.96 ± 0.6	48.96 ± 0.24	49.96 ± 0.32
**MCV pg**	15–18	16.06 ± 0.3	15.6 ± 0.2	15.76 ± 0.15	16.42 ± 0.2
**MCHC g/dL**	33.7–36.4	32.82 ± 0.5	32.54 ± 0.23	32.2 ± 0.4	32.88 ± 0.4
**Platelets 10^3^/µL**	674–1675	535.4 ± 139.7	582.4 ± 132	770.2 ± 189	661.8 ± 160.5

*n* = 5, mean counts are presented.

**Table 2:** Plasma Biochemistry of ALOS4 treated ICR mice.

	Normal Range	Saline	ALOS4 30 mg/kg	ALOS4 90 mg/kg	ALOS4 180 mg/kg
**Creatinine mg/dL**	0.2–0.4	0.31 ± 0.03	0.26 ± 0.014	0.25 ± 0.06	0.27 ± 0.02
**Calcium mg/dL**	9.8–10.8	11.34 ± 0.25	10.29 ± 0.11	10.38 ± 0.29	10.42 ± 0.1
**Phosphorus mg/dL**	6.4–11.3	10.43 ± 0.9	8.6 ± 0.56	9.53 ± 0.4	8.44 ± 0.75
**Glucose mg/dL**	169–282	176.2 ± 7.4	169.8 ± 6.9	170.75 ± 10.7	183.2 ± 14.3
**Urea mg/dL**	39–62	55.48 ± 2.9	46.08 ± 1.7	48.23 ± 3.2	46.02 ± 3.9
**Cholesterol mg/dL**	56–133	140.4 ± 7.9	108.2 ± 9.5	142.25 ± 13.7	141.8 ± 9.1
**TP g/dL**	4.7–5.8	6.23 ± 0.11	6.17 ± 0.18	6.32 ± 0.13	6.28 ± 0.09
**Alb g/dL**	3.3–4.0	4.3 ± 0.09	4.3 ± 0.14	4.3 ± 0.06	4.36 ± 0.024
**Globulin g/dL**	1.4–2.0	1.93 ± 0.12	1.87 ± 0.07	2.02 ± 0.07	1.59 ± 0.4
**Total Bilirubin mg/dL**	0.16–0.32	0.1 ± 0.02	0.11 ± 0.02	0.14 ± 0.02	0.11 ± 0.03
**Alkaline Phos IU/L**	43–125	0.31 ± 0.03	0.26 ± 0.014	0.25 ± 0.06	0.27 ± 0.02
**SGOT IU/L**	69–191	11.34 ± 0.25	10.29 ± 0.11	10.38 ± 0.29	10.42 ± 0.1
**SGTP IU/L**	26–120	10.43 ± 0.9	8.6 ± 0.56	9.53 ± 0.4	8.44 ± 0.75
**Sodium mmol/L**	151–156	176.2 ± 7.4	169.8 ± 6.9	170.75 ± 10.7	183.2 ± 14.3
**Potassium mmol/L**	7.3–10.2	55.48 ± 2.9	46.08 ± 1.7	48.23 ± 3.2	46.02 ± 3.9
**Chloride mmol/L**	110–119	140.4 ± 7.9	108.2 ± 9.5	142.25 ± 13.7	141.8 ± 9.1

*n* = 5, mean counts are presented.

## Data Availability

All data are provided as figures and tables and included in this paper.
